# The effects of DNA methylation and histone deacetylase inhibitors on human papillomavirus early gene expression in cervical cancer, an *in vitro *and clinical study

**DOI:** 10.1186/1743-422X-4-18

**Published:** 2007-02-26

**Authors:** Erick de la Cruz-Hernández, Enrique Pérez-Cárdenas, Adriana Contreras-Paredes, David Cantú, Alejandro Mohar, Marcela Lizano, Alfonso Dueñas-González

**Affiliations:** 1 Unidad de Investigación Biomédica en Cáncer, Instituto de Investigaciones Biomédicas, UNAM/Instituto Nacional de Cancerología (IIB, UNAM/INCan), Mexico City, Mexico; 2 Department of Gynecology, INCan, Mexico City, Mexico

## Abstract

**Background:**

The methylation status at the human papilloma virus (HPV) genome found in pre-invasive and invasive cervical lesions suggests that neoplastic transformation can be suppressed by gene hypermethylation, whereas hypomethylation accompanies or causes cancer progression; hence, epigenetic therapy aimed at reactivating cellular suppressor-gene expression has the potential to act as a tumor promoter by enhancing HPV oncoprotein expression in HPV-related malignancies. The objective of this study was to determine the influence of hydralazine and valproate on HPV oncogene expression in cervical cancer cell lines and the primary tumors of patients undergoing treatment with hydralazine and valproate.

**Results:**

Overall, hydralazine and valproate either alone or combined exerted a growth inhibitory effect on cervical cancer cell lines. A cell line-specific up-regulating effect was observed on E6/E7 gene expression, which in general correlated with DNA hypomethylation and histone acetylation at the long control region (LCR). Nonetheless, E6/E7 expression was unchanged or decreased in the majority of patients with cervical cancer treated with hydralazine, valproate, or both. In some cervical cancer cell lines, these drugs led to increased transcription of p53, and increased its stabilization due to acetylation at lysines 273 and 282, which allowed a higher bax-protein transactivating effect.

**Conclusion:**

The results of this study demonstrate that hydralazine and valproate can be safely administered to HPV-related malignancies such as cervical cancer because they do not increase viral oncoprotein expression. Most importantly, the antitumor effect of hydralazine and valproate in cervical cancer may at least partially depend on an up-regulating effect on p53 gene and on the valproate-induced hyperacetylation of p53 protein, protecting it from degradation by E6.

## Background

Cervical cancer a tumor related to high-risk human papillomavirus (HPV) infection remains as one of the greatest killers of women worldwide, particularly in underdeveloped countries [1]. The realization that viral infections, by insertion of viral genes into host genomes, can trigger host defense mechanisms such as methylation machinery activation has aroused interest in the study of the epigenetic events occurring in both virus and host genomes [2]. Human genomes harbor DNA sequences resembling retroviral long terminal repeats and the transposable elements, and indeed there are indications that under certain situations inappropriate "activation" of these normally silenced sequences could play a role in the carcinogenic process [3]. In addition, it has also been established that some viruses can find ways to adapt different tactics for regulating expression of their genes through modulation of DNA methylation; thus, a virus may silence activation of its genes in a manner that favors establishment of persistent infection and host immune defense evasion [4]. In addition, viral oncoproteins can possess the ability to modulate directly or indirectly the methylation machinery in order to silence cellular genes that could interfere with its tumor-promoting actions, this recently proven by Burgers et al., who demonstrated that E7 binds and stimulates the enzymatic activity of dnmt1 [5]

One of the first indications of the importance of DNA methylation and viral gene expression came from studies of cell transfection with HPV-16 *in-vitro *methylated genomes, demonstrating that under these circumstances DNA is transcriptionally repressed [6]. SiHa and CasKi cell lines that harbor HPV-16 and that have a couple of, and multiple, viral genome copies, respectively, possess a conserved profile of CpG hypo- and hypermethylation. Hypermethylation was found in genomic segments overlying late genes, while the long control region and the E6 and E7 oncogenes were unmethylated in SiHa cells. Evaluation of smears of normal, precursor, and invasive lesion smears shows that as lesion severity increases, there is progressive hypomethylation in LCR and E6 gene regions. These findings led authors to postulate that neoplastic transformation can be suppressed by gene hypermethylation, whereas hypomethylation accompanies or causes cancer progression [7]. Contrariwise, a study performed in HPV-18 cervical cancer cell lines HeLa and C4-1 and clinical samples found clonal heterogeneity in the methylation status of the different regions analyzed [8]. A study focused on the methylation of E2, the early gene that contributes to multiple biological processes including viral transcription and viral DNA replication, showed that the ability of E2 protein to bind E2 binding sites (E2BS) *in vitro *is inhibited by the methylation of these cytosines [9]. These observations may indicate that the methylation state of the viral genome, and particularly that of E2BSs, may vary during the viral life cycle, providing a novel means for modulating E2 function as infection progresses [10].

On the other hand, aberrant gene transcription resulting from epigenetic changes are frequent events in the molecular pathogenesis of malignant transformation. DNA hypermethylation and histone deacetylation are critical for determining a closed chromatin structure responsible for or related with aberrant gene transcription in malignancies [11]. In contraposition to genetic defects, the reversible nature of epigenetic aberrations constitutes an attractive therapeutic target; hence, a number of inhibitors of DNA methylation and histone deacetylases (HDAC) are at present undergoing pre-clinical testing in combination for cancer therapy [12-18]. Nevertheless, some authors argue for caution in the use of epigenetic therapy for HPV-related malignancies, because it may act as a tumor promoter by enhancing HPV oncoprotein expression.

Our group has been interested in the clinical development of hydralazine and valproate a DNA methylation and histone deacetylases (HDAC) inhibitors respectively as epigenetic therapy for cancer including cervical carcinoma [19-24]. Thus, we wanted to determine the influence of these drugs on HPV oncogene expression in cervical cancer cell lines and on the primary tumors of patients undergoing treatment with hydralazine and valproate.

## Results

### Growth inhibition by hydralazine and valproate acid in cervical cancer cells

**Figure 1:**
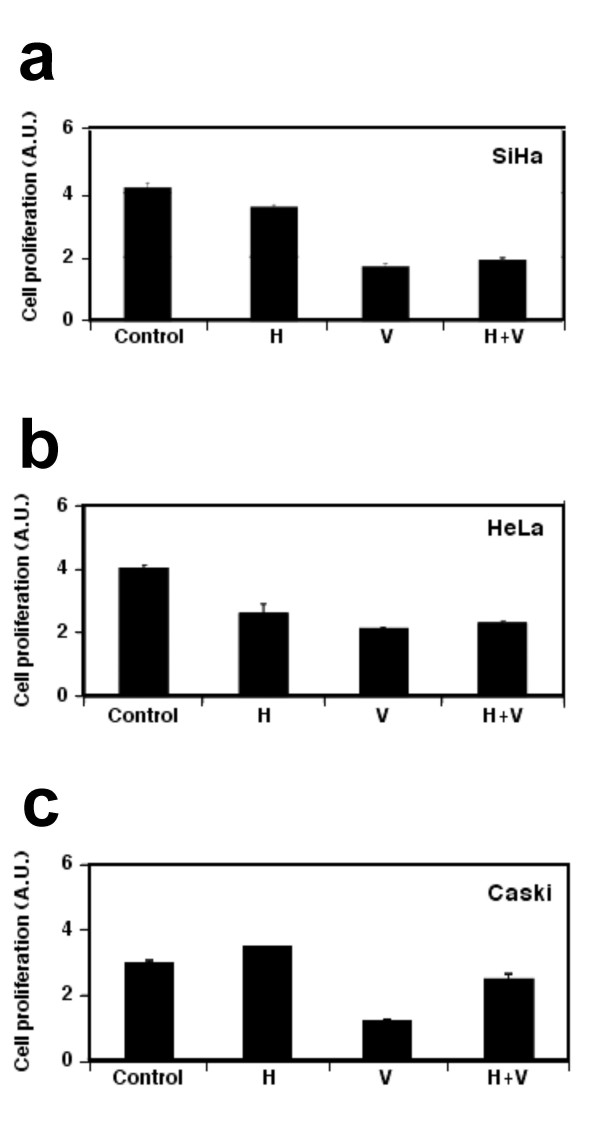
**Growth inhibition assays in cervical cancer cell lines**. Cells were cultured in the presence of hydralazine, valproate, and both drugs for 48 h; cell viability was measured at 72 h. Hydralazine exerted an inhibitory effect on HeLa, moderate inhibition on SiHa, and a minor stimulatory effect on CasKi cells. Valproate showed a consistent inhibition on the three cell lines.

As the initial approach to investigate the possible growth-promoting effect of hydralazine and valproate on HPV-positive cervical cancer cell lines, SiHa, CasKi, and HeLa were cultured in the presence of hydralazine, valproate, and both drugs for 48 h; cell viability was measured at 72 h. As showed in Figure 1, hydralazine exerted an inhibitory effect on HeLa cells and moderate inhibition on SiHa, and actually had a minor stimulatory effect on CasKi cells; on the contrary, valproate showed a consistent inhibition on the three cell lines. This effect was unchanged in SiHa and HeLa cells with both drugs in combination, whereas in CasKi the overall effect was inhibitory although less so than with valproate alone.

### E6/E7 gene expression in cervical cancer cell lines

**Figure 2:**
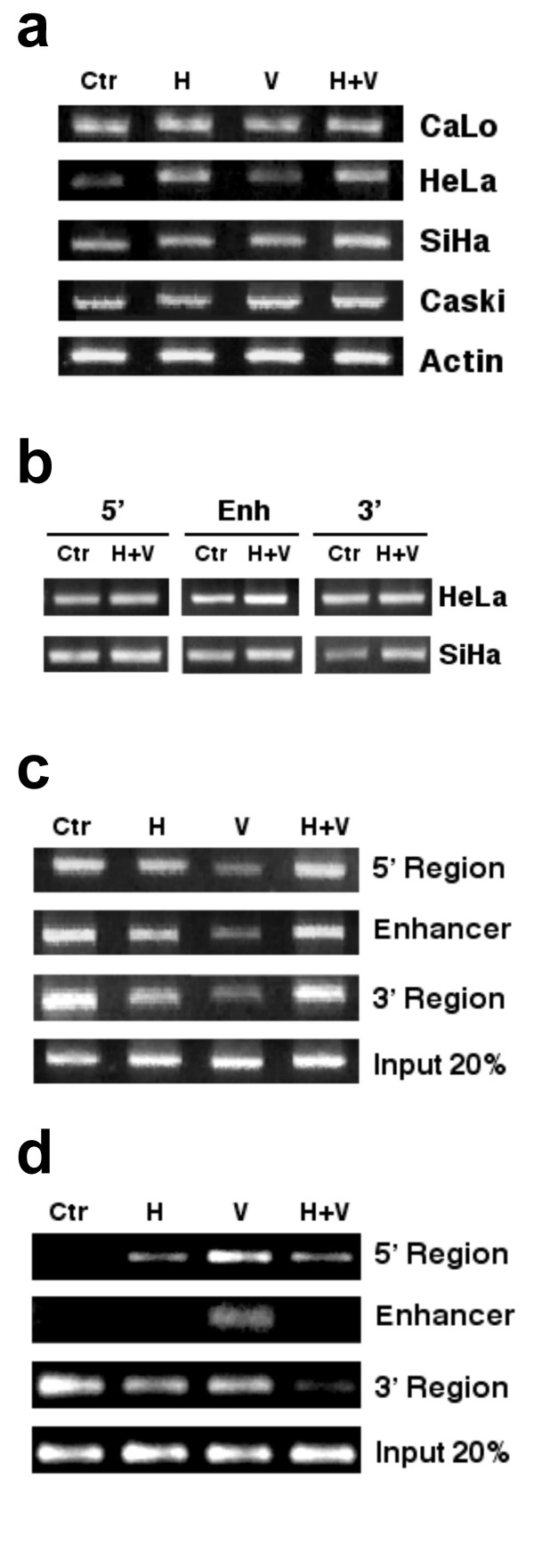
**Expression of E6/E7, methylation at LCR and ChIP assays**. **2a**. E6/E7 expression was assayed by RT-PCR. CaLo cells no changes in expression. In HeLa, hydralazine and hydralazine/valproate up-regulated the transcript 1.17- and 2.38-fold, respectively. In SiHa cells only the combination of hydralazine/valproate led to a 0.5-fold increase, while in CasKi, hydralazine, valproate, and both increased expression by 0.52-, 0.67-, and 0.83-fold, respectively. **2b**. Hydralazine/valproate led to a small increase in the intensity of the bands after digestion with the enzyme McrBC in the three LCR regions analyzed as compared with untreated cells, indicating a certain degree of hypomethylation. **3c**. ChIP assays with anti acetylated H4. In the CaLo, acetylated H4 was detected in the three regions analyzed; hydralazine, valproate, and both decreased the enrichment for acetylated H4. In line with increased E6/E7 expression in SiHa had an enrichment of acetylated H4 at the 5' region of the LCR, whereas only valproate affected this at the enhancer region.

To assess whether these epigenome targeting drugs could influence viral oncoprotein expression, E6/E7 transcript expression was assayed by RT-PCR in HPV18-positive CaLo and HeLa cell lines and in HPV16-positive CasKi and SiHa cells. As shown in Figure 2a, CaLo cells displayed essentially no changes in expression with either drug or with both drugs in combination as densitometric analyses showed a -0.1-fold variation. In HeLa cells, valproate induced no changes; however, hydralazine and hydralazine/valproate up-regulated the transcript 1.17- and 2.38-fold, respectively. With regard to HPV16 cell lines, in SiHa cells only the combination of hydralazine/valproate led to a 0.5-fold increase, while in CasKi, hydralazine, valproate, and both increased expression by 0.52-, 0.67-, and 0.83-fold, respectively.

### Methylation of the LCR in HeLa, and SiHa

To further correlate the transcriptional effect observed with LCR methylation status, the combined treatment of hydralazine/valproate was assessed in these cell lines. As expected, treatment led to a small increase in the intensity of the bands after digestion with the enzyme McrBC in the three LCR regions analyzed as compared with untreated cells, indicating a certain degree of hypomethylation (Fig 2b). These changes – although small – are nonetheless in line with the slightly higher E6/E7 transcript expression in these cell lines when treated with these drugs.

### H4 acetylation in Calo, CasKy, and HeLa cell lines

To further investigate whether changes in E6/E7 transcription are also associated with acetylated H4, chromatin immunoprecipitation experiments were performed in these cell lines, amplifying the entire LCR region in three amplicons: the 5'region; the enhancer region, and the 3'region. In the CaLo cell line, whose E6/E7 expression did not change with any of the treatments (Figure 1), acetylated H4 was detected in the three regions analyzed; however, hydralazine, valproate, and both actually led to a lower enrichment for acetylated H4, which may explain the lack of effect of these drugs on E6/E7 expression (Figure 2c). In line with increased E6/E7 expression in SiHa (Figure 1), hydralazine, valproate, and the combination led to an acetylated H4 enrichment at the 5'region of the LCR, whereas only valproate affected this at the enhancer region. (Figure 2d).

### Expression of E6/E7 in the primary tumors of patients treated with hydralazine, valproate, and hydralazine/valproate

**Figure 3:**
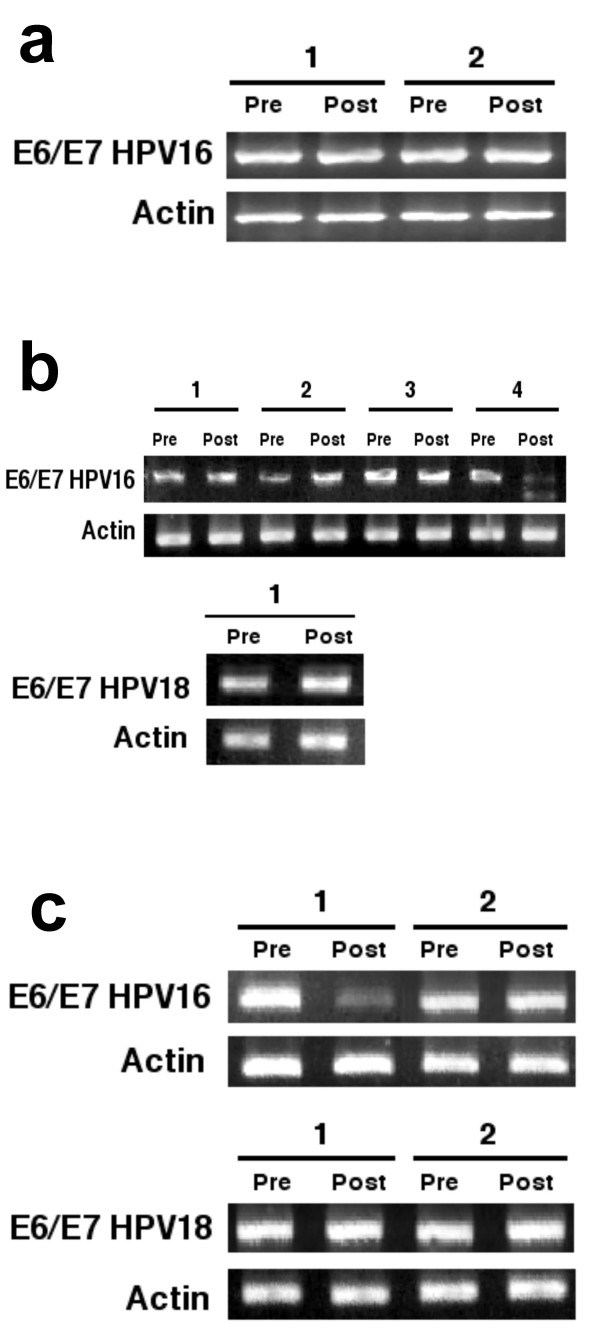
**E6/E7 expression in primary tumors**. 3a) shows the samples of two patients treated with hydralazine whose transcripts were unmodified with the treatment. 3b shows four HPV16 tumors of patients treated with valproate. There were no changes in two (1 and 3), a 0.74-fold increase in one (2), and a 0.7-fold decrease in another (4). In the HPV18 sample expression did not change. 3c) shows two HPV16 and two HPV18 tumors. A case (1) decreased with treatment while the remaining three had no changes.

To date, the results in cell lines showed heterogeneous treatment results with the epigenetic drugs, although in general these showed correlation between E6/E7 expression with DNA demethylation and histone hyperacetylation. To investigate whether these *in vitro *changes could also be observed in patients with cervical cancer treated with these drugs, we analyzed E6/E7 transcript level in the primary tumors of patients treated in three different clinical trials. Regarding patients treated with hydralazine in a phase I clinical trial already reported [22], only the pre- and post-treatment biopsies of two patients whose tumors harbored HPV16 were available for analysis. As can be seen in Figure 3a, no changes were observed; nonetheless, there was no availability of HPV18 samples. Regarding the valproate trial [24], four pairs of HPV16 and one carrying HPV18 tumor samples were available for analysis. As shown in Figure 3b, for the HPV16 samples we observed no changes at the transcript level in two, a 0.74-fold increase in one, and a 0.7-fold decrease in another, whereas in the HPV18 sample expression did not change (a 0.03-fold decrease). Finally, four cases were able to be analyzed in patients participating in a phase II trial of hydralazine and valproate plus cisplatin chemoradiation (*ClinicalTrials.gov Identifier: NCT00404326*). In this trial, 17 patients were treated. Hydralazine and valproate were started 7 days prior to the first application of cisplatin and radiation, and biopsies were taken at day 7; thus, no confounding effect of chemoradiatiation occurred. For this analysis, only four pairs of samples were available: two each of HPV16 and -18. As shown in Figure 3c, in one HPV16 tumor E6/E7 expression diminished (0.63-fold), whereas the other exhibited no change; expression was unchanged in both HPV18 samples. Taken together, E6/E7 expression in primary tumors did not change with treatment in eight, decreased in two, and increased in one (two hydralazine, five valproate, and four hydralazine/valproate) of 11 patients analyzed.

### p53 expression

**Figure 4:**
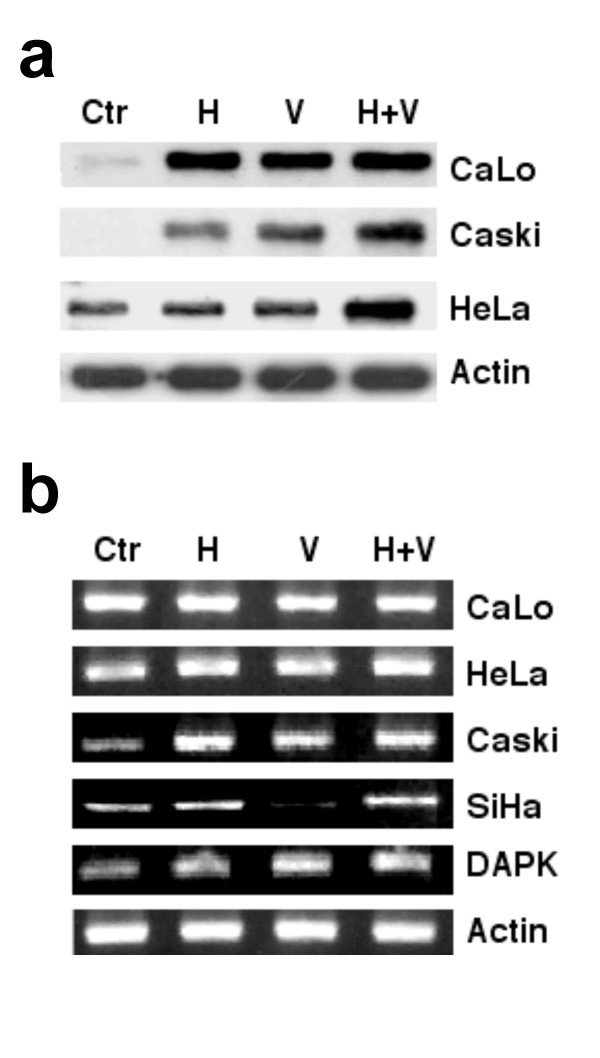
**p53 expression in cell lines**. Western blot (4a) and RT-PCR (4b) analyses of p53. Hydralazine, valproate and both increased p53 in CaLo and CasKi cells whereas only the combination did in HeLa cells. CaLo cells showed no transcriptional changes. p53 expression was increased between 1- and 2-fold in CasKi, whereas on slightly in HeLa. DAPK expression was increased as expected with any of these treatments.

Taken together, these results indicated that the epigenetic drugs investigated possessed no influence on HPV-early gene expression by tumor cells in a clinical scenario, despite the fact that *in vitro *treatment with these drugs in some cell lines led to a small increase in expression that correlated with H4 acetylation and LCR demethylation. Because we have observed up-regulation of p53 at the transcriptional level with the combination of hydralazine and valproate in tumors of patients with breast cancer [25], and in addition, histone deacetylase inhibitors acetylate non-histone transcription regulatory proteins [26], we wanted to analyze whether these epigenetic drugs would induce changes at p53, which is a key player in HPV carcinogenesis. For this, we first observed p53 protein levels by Western blot in CaLo, CasKi, and HeLa cells. As shown in Figure 4, there was a clear increase of p53 in CaLo and CasKi cells by hydralazine, valproate, and both, while in HeLa cells only the combination of hydralazine/valproate did. To investigate whether the p53 change occurs at the level of transcription, we analyzed its expression by RT-PCR. In CaLo, there were no transcriptional changes; nevertheless, in CasKi p53 expression increased between 1- and 2-fold, whereas in HeLa the effect was rather modest, with increased band intensities -0.3-fold. SiHa cells had p53 increased only by hydralazine and hydralazine/valproate. It is noteworthy that the transcriptional effect on p53 of hydralazine, valproate, and the combination in CasKi cells was even higher than that on the gene DAPK known to be transcriptionally silenced and reactivated with these drugs in this cell line.

### p53 acetylation

**Figure 5:**
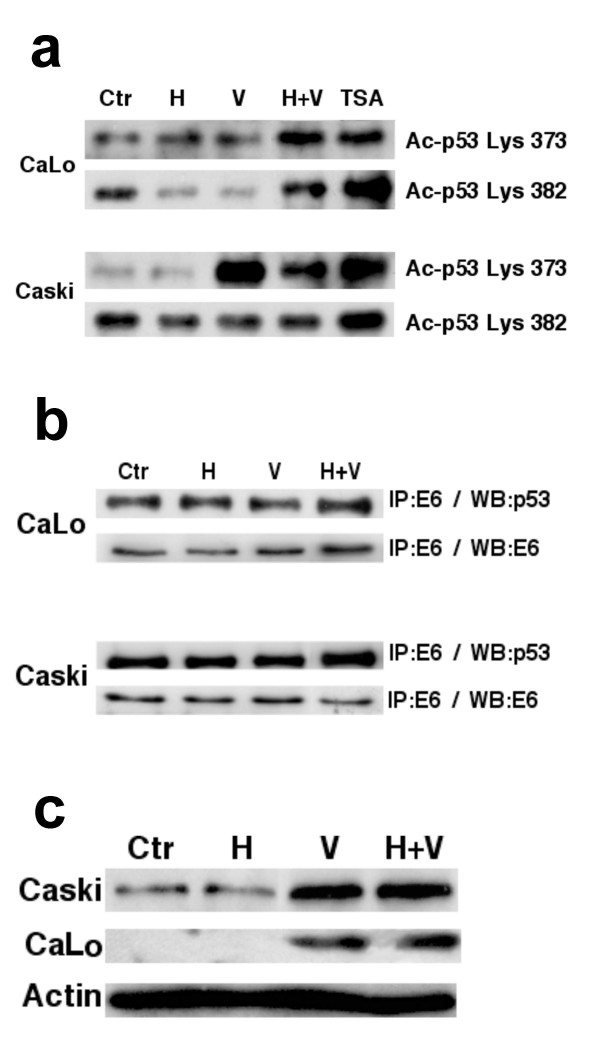
**p53 acetylation**. **5a**. Immunoprecipitation assays showing that the three drugs or the combination hyperacetylated p53 at lysine 373 whereas only the combination of hydralazine-valproate did at lysine 382 in CaLo cells. Lysine 373 was hyperacetylated in Caski by the combination. **5b**. Interaction by immunoprecipitation with E6 antibody and then Western blotted with a p53 antibody showing an increased p53 indicating that acetylation of p53 protects its degradation by E6. **5c**. Transactivation effect o p53 upon bax protein. Hydralazine and valproate in combination led to a strong increase in bax protein.

To date, these results indicate that changes at p53 occur at the protein, RNA, or both levels depending on the cell line. Because acetylation of p53 leads to an increased half-life that would explain changes observed in Western blots, we analyzed p53 acetylation by valproate alone and in combination with hydralazine, using trichostatin A as positive control. As shown in Figure 5a, immunoprecipitation assays showed that the three drugs or the combination hyperacetylated p53 at lysine 373 whereas only the combination of hydralazine-valproate did at lysine 382 in CaLo cells. Likewise, lysine 373 was hyperacetylated in Caski by the combination. It is of note that in this cell line, only TSA acetylated lysine 382. It has already demonstrated that the binding site of p53 for E6 encompasses the region containing lysines 372 and 382, and that their acetylation inhibits p53 ubiquitination induced by E6; thus, we analyzed this interaction by immunoprecipitating with E6 antibody and then Western blotted with a p53 antibody. Immunoprecipitates were also probed against E6 antibody as control. As shown in Figure 5b, despite the fact that valproate, valproate/hydralazine, and TSA slightly increased E6 level in these cells, p53 was also increased. These results indicate that acetylation of p53 protects its degradation by E6. Finally, to assess whether increased levels of p53 protein was functional, we evaluated in CaLo and CasKi cells the levels of bax protein, known to be transactivated by p53. As shown in Figure 5c, hydralazine and valproate in combination led to a strong increase in bax protein.

## Discussion

Currently, epigenetic therapy for malignant neoplasms is being evaluated in a number of malignancies using DNA methylation and HDAC inhibitors either alone or in combination with the aim of reactivating the expression of aberrantly silenced tumor suppressor genes [25,27-29]. Nonetheless, some authors have argued for caution in the use of epigenetic therapy for HPV-related malignancies as it may act as a tumor promoter by enhancing HPV oncoproteins expression. In this study, by analyzing the effect of the DNA methylation inhibitor hydralazine and the HDAC inhibitor valproate on cervical cancer cell lines and primary tumors of patients with cervical cancer treated with these drugs we demonstrate that although these drugs *in vitro *may increase oncoprotein gene expression, which correlates with demethylation and histone acetylation, in patients E6 and E7 transcripts are mainly unchanged. In addition, these drugs may exert a favorable effect on tumors because cell lines show up-regulation of p53 expression and acetylation of this protein that is key in cervical carcinogenesis.

The epigenetic-mediated silencing of cellular genes in cervical cancer is well-established and contributes to malignant progression [30,31]. In HPV carcinogenesis regulation of E6/E7 expression, the main viral oncogenes principally depend on viral E2 protein and several cellular transcription factors such as NF1, AP1, KRF1, Oct1, SP1, and YY1, and the glucocorticoid receptor [32-34]; therefore, it is yet unknown to what extent epigenetics contributes in this regulation. Results of this study reveal that DNA methylation and histone acetylation possess a heterogeneous participation, because in some cell lines neither of these inhibitors changed the expression while in others the methylation inhibitor, the HDAC inhibitor, or both produced a rather modest transcriptional effect on E6/E7. Nevertheless, the correlation found between DNA hypomethylation at three different LCR regions as evaluated by McrBC scanning and H4 hyperacetylation as a result of the combined treatment with hydralazine and valproate support that at least in some cervical cancer cell lines, epigenetic phenomena are active players in E6/E7 oncogene transcriptional regulation. These results are in agreement with those found in a previous study in which CasKi cells, known to have the majority of the integrated HPV DNA copies transcriptionally inactivated, were treated with the DNA methylation inhibitor 5-azacytidine. The study demonstrates that 5-azacytidine induces an increase in the intensity of the existing RNA messages and in the appearance of new transcripts in treated versus untreated; in contrast, trichostatin A fails to modulate viral transcription in this cell line [35].

The most important findings, however, comprise those encountered in the primary tumors of patients treated with these drugs. As opposed to cell lines, only one of 11 patients had a 0.7-fold increase in HPV16 E6/E7 transcription level, whereas there was a decrease in two cases. The majority (eight of 11), nonetheless, demonstrated no changes in the transcription of these viral oncogenes. This finding further underscores the complexity of HPV transcriptional regulation during carcinogenesis. These data strongly suggest that the epigenetic "status" in cancer cell lines and tumors are the consequence – rather than the cause – of the transcriptional activity of E6/E7 oncogenes; thus, epigenetic therapy agents cannot facilitate increased viral oncogene activation-driven tumor progression.

In cervical cancer, a number of experimental studies have consistently shown that although numerous events drive the malignant conversion of cervical carcinoma cells, E6/E7 expression appears to be continuously required to maintain their malignant phenotype [36-38]; in fact, removing E6 and E7 proteins from HeLa cells via a recombinant virus that expresses the bovine papillomavirus E2 protein led to growth inhibition and impaired activation of p53 and Rb pathways and repression of E2F-responsive genes inducing growth inhibitory signals to the cells [39]. Because hydralazine and valproate inhibit the growth of cervical cancer cell lines in studies and did not negatively affect their E6 and E7 expression, we wanted to investigate the changes at p53 that plays an important role in HPV carcinogenesis, on the basis that we had observed up-regulation of this tumor suppressor gene in patients with breast cancer on treatment with these drugs [25]. As shown in Figure 4, these drugs alone or in combination up-regulated p53 at RNA and protein levels depending on cell line. A direct transcriptional effect of epigenetic drugs on p53 has not been clearly demonstrated; however, demethylation in other genes influencing p53 expression, such as ASPP1 [40], could explain the results observed. On the other hand, hydralazine by a yet unknown mechanism may induce p53 [41], whereas valproic acid and other HDACs inhibitors also are known to induce p53 gene transcription [42-44]. The extent to which the transcriptional effect on p53 of hydralazine and valproate are determinant for the growth inhibitory effect observed in this study is unknown; nonetheless, the strongest influence on this gene may occur at the protein level as a consequence of valproic acid's acetylating effect at lysines 373 and 382. This is a novel finding regarding valproic acid, although it could be expected because depsipeptide and trichostatin A are reported to acetylate these lysines in p53 protein, this acetylation leading to its longer half-life due to a significant decrease in p53 ubiquitination [45,46]. It is known that oncogenic human papillomavirus types 16 and 18 utilize this cellular proteolytic system to target the tumor suppressor protein p53. The HPV E6 oncoprotein binds to a cellular protein of 100 kd, termed the E6-associated protein (E6-AP). The E6-E6-AP complex specifically interacts with p53, resulting in p53's rapid ubiquitin-dependent degradation [47,48]. The p53 binding site for the E6-E6-AP complex encompasses the region containing lysines 372 and 382; therefore, we analyzed whether acetylated p53 could be protected from degradation by E6. As shown in Figure 5, despite the fact that valproate, valproate/hydralazine, and TSA slightly increased E6 levels, p53 was also increased, indicating that p53 acetylation protects its degradation by E6 and the expected p53 was functional, as shown by strong transactivation on the p53-responsive gene bax [49].

## Conclusion

The results of this study demonstrate that epigenome-targeting drugs such as hydralazine and valproate used to reactivate the expression of tumor suppressor genes silenced by epigenetic aberrations, can be safely administered to HPV-related malignancies such as cervical cancer because these do not induce major changes in the expression of viral oncoproteins in patients treated with these drugs, this despite the fact that in at least some cervical cancer cell lines, hydralazine and valproate may cause a small up-regulating effect on E6/E7 oncogenes. Most importantly, the antitumor effect of hydralazine and valproate in cervical cancer may at least partially depend on an up-regulating effect on the p53 gene and in the valproate-induced hyperacetylation of p53 protein protecting it from its degradation by E6.

## Methods

### Cell culture

Cervical cancer cell lines Siha, CasKi, Hela, and Calo were cultured at 37°C in a humidified atmosphere containing 5% CO_2 _in DMEM supplemented with 10% (v/v) fetal calf serum (Life Technologies, Inc.)

### DNA extraction and LCR methylation

DNA derived from cell lines was purified with DNeasy Tissue Kit (Qiagen) following protocols suggested by the supplier. For McrBC digestion (New England Biolabs), 250 ng of DNA was digested with 3 U of enzyme for 1 h at 37°C in 25 μL of NE buffer 2 (50 mM NaCl, 10 mM Tris-HCl, 10 mM MgCl_2_, 1 mM dithiothreitols [pH 7.9]). Table 1 summarizes the primers employed for LCR methylation analysis. The PCR mixture contained 1× PCR buffer, 0.5 U of Taq Gold polymerase, dNTPs (each 1.25 mM), and 0.5 μL of DNA digested with McrBC in a final volume of 20 μL. Conditions were 95°C for 10 min followed by 30 cycles of 95°C for 30 sec, 59°C for 30 sec, and 72°C for 35 sec, with a final extension cycle of 72°C for 6 min. The PCR product was separated on a 2% agarose gel, stained with ethidium bromide, and visualized under ultraviolet (UV) illumination.

**Table 1:** Primers and conditions for amplification.

Primer Set	Sense 5' – 3'	Antisense 5' – 3'	Size, (pb)
HPV16 5' region	caccacctcatctacctc	caggatgtagcaaatatag	292
HPV16 Enhancer	ctatatttgctacatcctg	cagcggtatgtaaggc	319
HPV16 3' region	gccttacataccgctg	gctctgtgcataactgtgg	382
HPV18 5' region	cgtgccaggaagtaatatg	gatgctgtaaggtgtgcag	301
HPV18 Enhancer	ctgcacaccttacagcatc	gggtagacagaatgttgg	368
HPV18 3' region	ccaacattctgtctaccc	gttccgtgcacagatcag	266
HPV16 E6-E7	gggaatccatatgctgtatg	gggaagcttttatggtttctgagaacag	597
HPV18 E6-E7	gaatttgcattcaaagatttatttg	gggggaattcttactgctgggatgcacacc	673
p53	ctgaggttggctctgactgtaccaccatcc	ctcattcagctctcggaacatctcgaagcg	390

### Cytotoxicity assays

Cells were seeded into 96-well microtiter Falcon plates (Becton Dickinson, Franklin Lakes, NJ, USA) at 1.5–2.5 × 103 cells/well in 0.1 mL of complete medium. The following day, cells were treated for 5 days in complete medium with hydralazine at 10 μM and magnesium valproate at 1 mM. Medium with drugs was changed every other day. At day 6, cell viability was measured by conventional MTT dye reduction assay. Briefly, 50 μL of 5 mg/mL MTT reagent in PBS was added to each well. Viable cells with active mitochondria reduce MTT to an insoluble purple formazan precipitate that is solubilized by the subsequent addition of 150 μL of DMSO. The formazan dye was measured spectrophotometrically using an ELISA reader. All assays were performed in triplicate. The cytotoxic effect of each treatment was expressed as a percentage of cell viability relative to untreated control cells (percentage of control) and is defined as [(A570 nm-treated cells)/A570 nmnon-treated cells)] × 100.

### RNA extraction and expression analysis

Total RNA was obtained from the cell lines treated, or from frozen biopsies using RNeasy Mini kit (Qiagen) according to manufacturer instructions. Samples were treated with 1 U of DNAse I (Gibco-BRL). Amount of RNA was determined by UV spectrophotometry and quality was assessed in 2% agarose gels. For cDNA preparation, 1 μg of total RNA was inversely transcribed with random hexamers using the SuperScript First-strand synthesis system for RT-PCR (Invitrogen). The obtained cDNA was PCR-amplified for E6/E7 expression analysis. For DAPK gene, primers and PCR conditions used for RT-PCR were performed as described previously [22].

### Western blot

Total cellular proteins were extracted from cells harvested from a 75-cm^2 ^plate. Cells were pelleted and disrupted with 300 μL of a lysis buffer (100 mM Tris, pH 8, 100 mM NaCl, 0.5% Nonidet P-40, 1% apoprotein, 1 mM PMSF). Proteins were boiled in sample buffer (125 mM Tris-HCL, pH 6.8, 1% SDS, 2% β-mercaptoethanol, and 0.01% bromophenol blue) for 5 min and then loaded onto 10–18% SDS-PAGE. After electrophoresis, proteins were transferred onto a nitrocellulose Hybond-C extra membrane (Amersham) in a wet chamber during 1 h at 100 V. Membranes were then blocked with TBS 1× containing 1% skimmed milk and 0.1% Tween-20, washed, and incubated with the corresponding antibody (E6, p53, Bax, Actin) (Santa Cruz Biotechnology, Santa Cruz, CA, USA). Horseradish peroxidase-conjugated secondary antibody was used for protein-primary antibody complex detection. Levels of the corresponding proteins were visualized using the ECL system (Amersham).

### Immunoprecipitation

Cells were harvested and then lysed in lysis buffer (1% NP-40, 150 mM NaCl, 50 mM Tris, 0.05% SDS, 1 mM PMSF, and a 1% cocktail of protease inhibitors) on ice for 20 min. After centrifugation at 4°C at 13,000 rpm for 10 min, antibodies (p53 or E6; Santa Cruz Biotechnology) were added to the supernatant on ice for 1 h. Protein G-agarose (Santa Cruz Biotechnology) was then added to the samples, and the samples were rolled at 4°C for 1 h. After the beads were washed three times with lysis buffer, the pellets were dissolved into 2 μL of SDS loading buffer after centrifugation. The protein was analyzed by Western blotting with different antibodies: anti-E6 (Santa Cruz Biotechnology) or anti-acetylated p53 (Lys373 and -382; Upstate).

### ChIP analysis

Cells were treated with 1% formaldehyde at room temperature for 10 min under constant agitation. The reaction was stopped by the addition of glycine to a final concentration of 125 mM. Cells were washed twice in ice-cold PBS 1×, resuspended in lysis buffer (50 mM Hepes-KOH pH7.9, 10 mM EDTA pH 8.0, 1% SDS) containing protease inhibitors, and sonicated on ice until crosslinked chromatin was sheared to an average DNA fragment length of 0.5–1 kbp. After centrifugation, soluble crosslinked chromatin was diluted 1:10 in immunoprecipitation (IP) buffer (10 mM Hepes-KOH pH 7.9, 1% Triton X-100, 150 mM NaCl, and protease inhibitors) divided into aliquots and stored at -70°C. Protein A-Agarose (Upstate) was blocked with BSA (1 mg/mL) and Salmon-sperm DNA (Sigma-Aldrich) and in IP buffer for 4–6 h at 4°C and subsequently washed extensively with IP buffer before use. Chromatin preparations were pre-cleared by incubation with blocked protein A-agarose for 2 h at 4°C. The protein A-agarose was removed by centrifugation; the pre-cleared chromatin was incubated with antibody (anti acetyl-histone antibody, Upstate) for 12–14 h at 4°C. Immunoprecipitates were recovered by incubation with fresh blocked protein A-agarose for 2 h at 4°C, followed by low-speed centrifugation. The pellets were washed three or four times with IP buffer, three times in wash buffer (10 mM Tris-HCl pH 8.0, 0.25 mM LiCl, 0.5% NP-40, 0.5% sodium deoxycholate, 1 mM EDTA pH 8.0), and three times in Tris-EDTA (TE) pH 8.0. Precipitates were then extracted by incubation with elution buffer (50 mM Tris pH 8.0, 1% SDS, 50 mM NaHCO_3_, 1 mM EDTA pH 8.0), and formaldehyde crosslinks were reversed by treatment with a 1/25 volume of 5 M NaCl for 8 h at 65°C. The DNA was purified by extraction with phenol and precipitation with ethanol and analyzed by PCR with primers specific (Table 1). PCR products were separated on a 2% agarose gel and visualized by ethidium bromide staining.

## Competing interests

The author(s) declare that they have no competing interests.

## Authors' contributions

EC-H performed the majority of the experimental work; EP-C and AP-C contributed in the experimental work and analysis of results; AM contributed with the analysis and discussion of results. ML and AD-G conceived and wrote the manuscript.
